# The Role of Surinamese Migrants in the Transmission of *Chlamydia trachomatis* between Paramaribo, Suriname and Amsterdam, The Netherlands

**DOI:** 10.1371/journal.pone.0077977

**Published:** 2013-11-13

**Authors:** Reinier J. M. Bom, Jannie J. van der Helm, Sylvia M. Bruisten, Antoon W. Grünberg, Leslie O. A. Sabajo, Maarten F. Schim van der Loeff, Henry J. C. de Vries

**Affiliations:** 1 Public Health Laboratory, Cluster Infectious Diseases, Public Health Service of Amsterdam, Amsterdam, The Netherlands; 2 Department of Research, Cluster Infectious Diseases, Public Health Service of Amsterdam, Amsterdam, The Netherlands; 3 STI Outpatient Clinic, Cluster Infectious Diseases, Public Health Service of Amsterdam, Amsterdam, The Netherlands; 4 Department of Experimental Virology, Academic Medical Center, University of Amsterdam, Amsterdam, The Netherlands; 5 Lobi Foundation, Paramaribo, Suriname; 6 Dermatological Service, Ministry of Health, Paramaribo, Suriname; 7 Department of Dermatology, Academic Medical Center, University of Amsterdam, Amsterdam, The Netherlands; 8 Center for Infections and Immunity Amsterdam (CINIMA), Academic Medical Center, University of Amsterdam, Amsterdam, The Netherlands; 9 Center for Infectious Disease Control, National Institute of Public Health and the Environment, Bilthoven, The Netherlands; University of California, San Francisco, University of California, Berkeley, and The Children's Hospital Oakland Research Institute, United States of America

## Abstract

The large Surinamese migrant population in the Netherlands is a major risk group for urogenital *Chlamydia trachomatis* infection. Suriname, a former Dutch colony, also has a high prevalence of *C. trachomatis*. Surinamese migrants travel extensively between the Netherlands and Suriname. Our objective was to assess whether the Surinamese migrants in the Netherlands form a bridge population facilitating transmission of *C. trachomatis* between Suriname and the Netherlands. If so, joint prevention campaigns involving both countries might be required.

Between March 2008 and July 2010, participants were recruited at clinics in Paramaribo, Suriname and in Amsterdam, the Netherlands. Participants were grouped as native Surinamese, native Dutch, Surinamese migrant, Dutch migrant, or Other, based on country of residence and country of birth of the participant and of their parents. Risk behavior, such as sexual mixing between ethnic groups, was recorded and *C. trachomatis* positive samples were typed through multilocus sequence typing (MLST). A minimum spanning tree of samples from 426 participants showed four MLST clusters. The MLST strain distribution of Surinamese migrants differed significantly from both the native Surinamese and Dutch populations, but was not an intermediate state between these two populations. Sexual mixing between the Surinamese migrants and the Dutch and Surinamese natives occurred frequently. Yet, the MLST cluster distribution did not differ significantly between participants who mixed and those who did not.

Sexual mixing occurred between Surinamese migrants in Amsterdam and the native populations of Suriname and the Netherlands. These migrants, however, did not seem to form an effective bridge population for *C. trachomatis* transmission between the native populations. Although our data do not seem to justify the need for joint campaigns to reduce the transmission of *C. trachomatis* strains between both countries, intensified preventive campaigns to decrease the *C. trachomatis* burden are required, both in Suriname and in the Netherlands.

## Introduction

*Chlamydia trachomatis* infections occur endemically among the general population of the Netherlands, but most infections are found in defined risk groups, such as adolescents and men who have sex with men (MSM) [1]. In addition, some ethnic minority groups are affected disproportionally by *C. trachomatis* infections [2]. Migrants from Suriname constitute one of the largest ethnic minority groups in the Netherlands, and the highest prevalence of *C. trachomatis* has been reported in this population. In 2011, the *C. trachomatis* prevalence among clients of the Dutch sexually transmitted infection (STI) clinics was 18% for heterosexual Surinamese migrants compared with 11% for native Dutch heterosexuals [3].

Suriname is a former colony of the Netherlands in the Caribbean region. It is a multi-ethnic society consisting of Creoles, Maroons, Hindustani, Javanese, Chinese, Caucasians, and indigenous Amerindians, as well as a mixed race population. Although considered an upper middle-income country by World Bank standards, Suriname is an emerging economy in which reliable diagnostics to detect *C. trachomatis* infections and other STIs are still lacking for the majority of its inhabitants [4]. The prevalence of *C. trachomatis* infections is high; one study demonstrated around 10% prevalence in a low-risk population (clients of a birth control clinic) and 21% in a high-risk population (clients of an STI clinic) [5].

Since the independence of Suriname in 1975, a large proportion of the Surinamese population migrated to the Netherlands and today almost as many people of Surinamese origin live in the Netherlands as in Suriname itself. Amsterdam has the largest number of Surinamese inhabitants outside of Suriname. As a result, traveling between the two countries is common. A study in 2008 found that more than half of the population of Surinamese descent living in the Netherlands had visited friends and relatives in Suriname during the preceding five years [6]. Of these travelers, 9% reported unprotected sexual contact in both countries [2].

Discordant sexual mixing is defined as sex between partners from two different groups (e.g. with different age or ethnicity) [7]. A group characterized by a high degree of sexual mixing can act as a bridge population for STI transmission between seemingly unrelated groups. Surinamese migrants living in the Netherlands may thus be a bridge population for STI transmission between the native populations in Suriname and the Netherlands [6]. If this is the case, it has implications for the design of effective preventive measures to reduce STI transmission. Joint campaigns involving both countries and a focus on travelers might be needed to reduce overall STI prevalence and to increase the impact of prevention.

We hypothesized that Surinamese migrants constitute a bridge population for the transmission of *C. trachomatis* between Suriname and the Netherlands. If this were the case, this would be reflected by:

1. A high degree of sexual mixing with native Surinamese and native Dutch partners, and
2. A distribution of *C. trachomatis* genotypes that is more similar to the native Surinamese or native Dutch population among the Surinamese migrants who report sexual mixing with the native Surinamese or native Dutch population respectively, compared with those that do not report sexual mixing.

We collected *C. trachomatis* positive urogenital samples in the two capital cities: Paramaribo, Suriname; and Amsterdam, the Netherlands, and genotyped the *C. trachomatis* strains with a high-resolution multilocus sequence typing (MLST) method [8], [9]. This method was specifically designed to have a discriminatory power as required for molecular epidemiological analyses. It was epidemiologically validated on clinical samples to differentiate *C. trachomatis* strains on a population level in a previous study [8]. These MLST data were used to compose a minimum spanning tree and elucidate *C. trachomatis* strain clusters. In a previous study, this approach successfully demonstrated the distinct transmission of *C. trachomatis* strains in MSM and heterosexuals [9]. In this study, the distribution of clusters was related to predefined risk characteristics like sexual mixing between native and migrant populations in the two countries.

## Materials and Methods

### Study sites and population

Participants were recruited at two sites in Paramaribo, Suriname and at one site in Amsterdam, the Netherlands:

1. The Dermatological Service in Paramaribo, an integrated outpatient clinic that offers free-of-charge examination and treatment of STIs and infectious skin diseases such as leprosy and leishmaniasis. Recruitment took place between March 2008 and July 2010.
2. The Lobi Foundation, a center for birth control and sexual health in Paramaribo. Recruitment took place between July 2009 and April 2010.
3. The STI Outpatient Clinic of the Public Health Service of Amsterdam, which is a low threshold clinic serving over 30,000 clients annually. Individuals were prioritized based on a short questionnaire to estimate the risk of having an STI [10]. Those considered at high-risk of an STI were eligible to participate. Recruitment took place between November 2009 and May 2010.

Exclusion criteria were: age younger than 18 years, antibiotic use in the previous 7 days, men having sex with men in the past 6 months, and previous participation in this study. After written informed consent, participants were given a unique code to participate anonymously. Participants were interviewed about demographic characteristics, including place of birth, place of birth of both parents, ethnicity of sexual partners, number of sexual partners, and place of residence of their sexual partners. The ethics committees of the Ministry of Health of the Republic of Suriname (VG010-2007) and the Academic Medical Center, University of Amsterdam, the Netherlands (MEC07/127) approved the study. Part of the data collected in the Netherlands has been described previously by *Bom et al.* (2013) [9]. Part of the data collected in Suriname has been described previously by *Van der Helm et al.* (2012) [5].

### Sample collection

In Paramaribo, urine samples from men and nurse-collected vaginal swabs were obtained, shipped and tested with the Aptima *Chlamydia* assay for the detection of *C. trachomatis* rRNA (Hologic Gen-Probe Inc., San Diego, USA) at the Public Health Laboratory in Amsterdam. The urine samples were tested within 40 days, and the vaginal swabs within 50 days after collection. A more detailed description has been reported in a previous study [5]. In Amsterdam, urine samples from men and nurse-collected vaginal or cervical swabs were tested with the Aptima assay at the Public Health Laboratory in Amsterdam. For each individual, only one *C. trachomatis* positive sample was selected. For female participants, vaginal samples were preferred; if vaginal samples were not available, cervical samples were selected. A more detailed description has been reported in a previous study [9].

### MLST

Nucleic acids from *C. trachomatis*-positive clinical samples were extracted and tested for the presence of chlamydial DNA [11], [12]. DNA isolates were amplified by a nested PCR and sequenced for the regions *ompA*, CT046 (*hctB*), CT058, CT144, CT172, and CT682 (*pbpB*) [8], [9]. The sequences were checked against the *Chlamydia trachomatis* MLST database (mlstdb.bmc.uu.se). Only samples of which all 6 loci were successfully amplified, sequenced, and identified, and therefore had obtained a full sequence type (ST) or MLST profile, were included in the analyses. As *ompA* is part of the MLST scheme, genovars could be assigned for all included samples. A minimum spanning tree was generated using MLST profiles. As the number of STs was too large to be used in statistical analyses, cluster analysis was performed, allowing single locus variance using BioNumerics 7.0 (Applied Maths, Sint-Martens-Latem, Belgium). A cluster was defined as a group of STs differing by not more than 1 locus from another ST within that group. Only clusters of at least 25 samples were included in the cluster analysis as these clusters were large enough for statistical analyses. The remaining samples were compiled in a residual group, which was used in the analyses as an additional cluster.

### Statistical analysis

**Figure 1:**
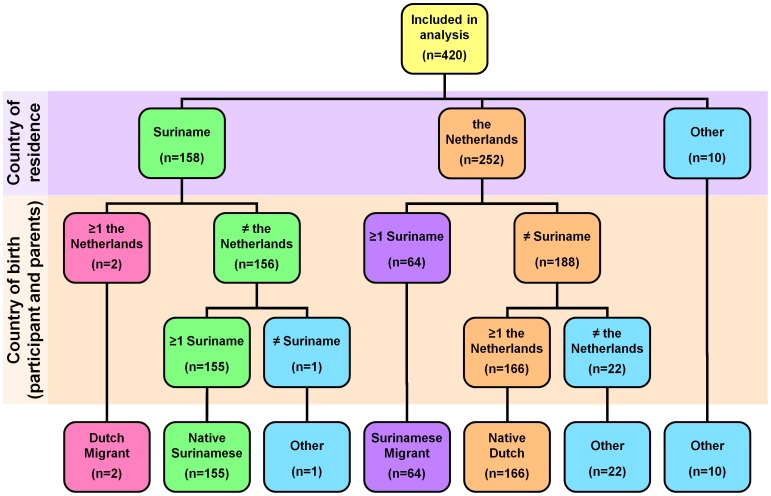
Algorithm for classification of participants into five groups (‘Native Surinamese,’ ‘Native Dutch,’ ‘Surinamese Migrant,’ ‘Dutch Migrant,’ and ‘Other’) based on country of residence of the participant and country of birth of the participant and his or her parents. Data were missing for 6 of the 426 participants, therefore they were not included in the analyses. The category ‘Other’ was derived from three different routes in the algorithm (n = 33).

Participants were classified into 5 groups based on country of residence of the participant, and country of birth of the participant, and of his or her parents (*Figure 1*):

1. *Native Surinamese group*: Participants residing in Suriname; neither the participant, nor the parents were born in the Netherlands, and at least one was born in Suriname;
2. *Native Dutch group*: Participants residing in the Netherlands; neither the participant, nor the parents were born in Suriname, and at least one was born in the Netherlands;
3. *Surinamese Migrant group*: Participants residing in the Netherlands; the participant and/or his or her parents (one or both) were born in Suriname;
4. *Dutch Migrant group*: Participants residing in Suriname; the participant and/or his or her parents (one or both) were born in the Netherlands;
5. *Other groups*: A participant neither residing in Suriname nor in the Netherlands; or neither the participant nor his or her parents were born in Suriname or the Netherlands.

Sexual partners in the past 12 months were classified into three groups based on country of residence of the sexual partner, and the ethnicity of the sexual partner, as perceived by the participant:

1. *Native Surinamese partner*: sexual partner residing in Suriname and of perceived Surinamese ethnicity;
2. *Native Dutch partner*: sexual partner residing in the Netherlands and of perceived Dutch ethnicity;
3. *Surinamese Migrant partner*: sexual partner residing in the Netherlands and of perceived Surinamese ethnicity.

A partner of perceived Surinamese ethnicity was defined as a partner with a Creole, Hindustani, Javanese, Chinese, Maroon, Amerindian, or mixed race ethnicity. A partner of perceived Dutch ethnicity was defined as a partner with a Caucasian ethnicity. Differences between clusters were tested using Pearson's *χ* ^2^ tests for categorical data. Fisher's exact tests were used when the expected cell count was less than one. For continuous data, Mann-Whitney *U* tests and Kruskal-Wallis tests were used. A *p*-value of <0.05 was considered statistically significant. Analyses were performed with SPSS package version 19.0 (SPSS Inc., Chicago, IL). To test whether the distribution of *C. trachomatis* clusters found within the Surinamese migrant population was an intermediate state between the distributions of the native Surinamese and native Dutch populations, a range of hypothetical intermediate cluster distributions was calculated. The proportion of every cluster within a hypothetical intermediate state (*p_hyp_i_*) was calculated by:
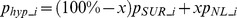
where *p_SUR_i_* is the proportion of cluster *i* in the native Surinamese population, *p_NL_i_* is the proportion of cluster *i* in the native Dutch population and *x* ranges from 0% to 100% with a step size of 1%. With *x* = 0%, the hypothetical distribution would be identical to the distribution found in the native Surinamese population, whereas *x* = 100% would result in a distribution identical to the one found among the native Dutch. To test whether these hypothetical intermediate distributions differed significantly from the distribution found among the Surinamese migrants, the proportions of each cluster were multiplied by the total number of Surinamese migrants and compared to the actual distribution of clusters found among the Surinamese migrants using Pearson's *χ^2^* tests.

## Results

### Study population and specimens

A total of 415 men and 1093 women participated in Paramaribo and 449 men and 1051 women participated in Amsterdam. Of those, 95 (23%) men and 129 (12%) women in Paramaribo and 100 (22%) men and 204 (19%) women in Amsterdam tested positive for *C. trachomatis*. A total of 508 (96%) *C. trachomatis* positive samples were available for further testing, of which 219 originated from Paramaribo and 289 from Amsterdam. In 450 samples (89%), the presence of genomic DNA could be demonstrated by qPCR. Of these 450 samples, 426 (95%) could be completely typed by MLST, of which 170 originated from Paramaribo and 256 originated from Amsterdam.

*Table 1* shows general characteristics of the 426 *C. trachomatis* positive participants, with a complete MLST profile by city. The median age of the participants in Paramaribo was higher (25 years; interquartile range (IQR): 22–30 years) compared with those from Amsterdam (23 years; IQR: 21–26 years; *p*<0.001). In addition, participants from Paramaribo usually had received less education (*p*<0.001) and reported fewer sexual partners (*p* = 0.011).

**Table 1:** General characteristics of *Chlamydia trachomatis*-positive men and women included in Paramaribo, Suriname and Amsterdam, the Netherlands, 2008–10.

		Paramaribo (n = 170)	Amsterdam (n = 256)	*p*
		n (%)	n (%)	
**Gender**	Male	65 (38)	86 (34)	**0.33**
	Female	105 (62)	170 (66)	
**Age in years**	Median (mean; IQR)	25 (27.0; 22–30)	23 (24.9; 21–26)	**<0.001**
**Education** a	Low	68 (41)	2 (1)	**<0.001**
	Medium	76 (46)	120 (48)	
	High	21 (13)	130 (52)	
**Ethnic group** b	Native Surinamese	155 (92)	-	**<0.001**
	Native Dutch	-	166 (66)	
	Dutch migrant	2 (1)	-	
	Surinamese migrant	4 (2)	60 (24)	
	Other	7 (4)	26 (10)	
**Number of sexual partners in the past 12 months** c	Median (mean; IQR)	1 (1.4; 1–2)	1 (2.0; 1–2)	**0.011**
***ompA*** ** genovar**	B	3 (2)	2 (1)	**0.59**
	D	31 (18)	29 (11)	
	E	55 (32)	101 (39)	
	F	33 (19)	52 (20)	
	G	9 (5)	16 (6)	
	H	2 (1)	2 (1)	
	I	22 (13)	31 (12)	
	J	10 (6)	13 (5)	
	K	5 (3)	10 (4)	
**MLST Cluster**	Cluster 1	43 (25)	62 (24)	**<0.001**
	Cluster 2	9 (5)	67 (26)	
	Cluster 3	37 (22)	25 (10)	
	Cluster 4	27 (16)	26 (10)	
	Residual group	54 (32)	76 (30)	

a *Data were missing for 5 participants recruited in Paramaribo and 4 in Amsterdam.*

b *Data were missing for 2 participants recruited in Paramaribo and 4 in Amsterdam.*

c *Data were missing for 5 participants recruited in Paramaribo and 1 in Amsterdam.*

IQR: interquartile range; MLST: multilocus sequence typing.

### Genovar distribution

As *ompA* is part of the MLST scheme, genovars could be assigned for all typed samples. In both cities we found 9 different genovars, being B, and D through K. The genovar distribution found in Paramaribo and Amsterdam was not significantly different (*p* = 0.59) with genovars D, E, F and I being most common (*Table 1*).

### MLST results

Among the 170 MLST profiles from Paramaribo there were 65 different STs (*Table S1*). Of these STs, 29 had multiple representatives (n = 2–28) and 36 were found in only a single isolate (singleton). Among the 256 Amsterdam samples, 124 different STs were found (*Table S1*). Multiple representatives were seen for 34 STs (n = 2–28) and 90 singletons were found. In the total population of the 2 cities, we identified 166 STs (n = 1–46) of which 106 were singletons.

**Figure 2:**
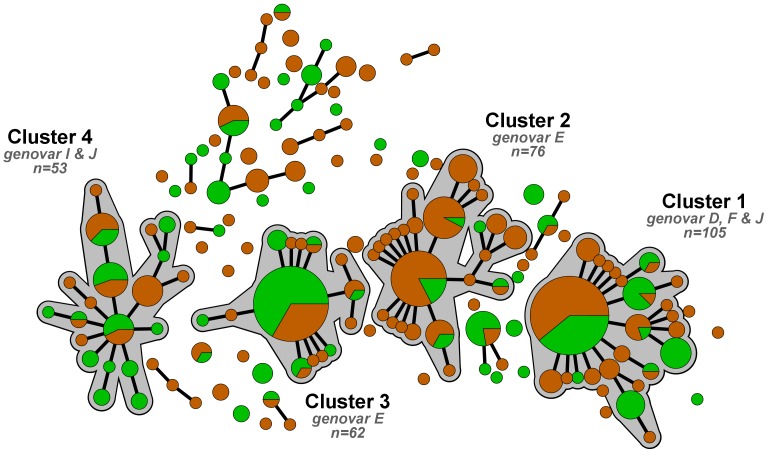
Minimum spanning tree of 426 *Chlamydia trachomatis*-positive samples from Amsterdam and Paramaribo, 2008–2010. Each circle represents one MLST type. Size of the circles is proportional to the number of identical MLST profiles. Bold lines connect types that differ by one single locus. Halos indicate clusters. Colors indicate city of sampling; green: samples from Paramaribo, Suriname (n = 170) and orange: samples from Amsterdam, the Netherlands (n = 256).

**Figure 3:**
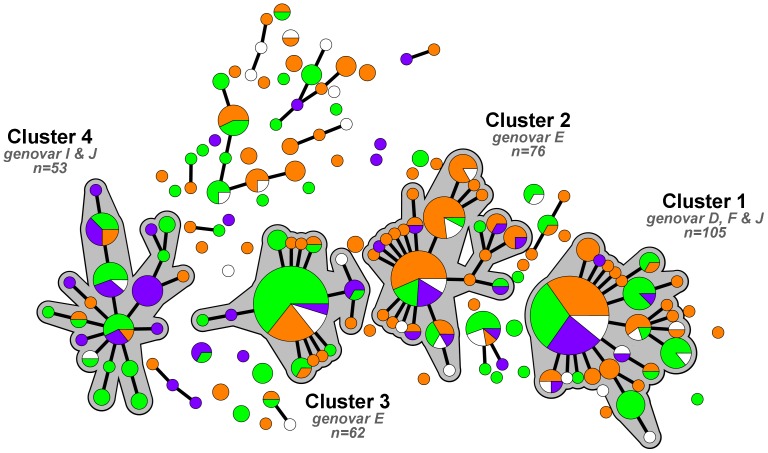
Minimum spanning tree of 426 *Chlamydia trachomatis*-positive samples from Amsterdam and Paramaribo, 2008–2010. Each circle represents one MLST type. Size of the circles is proportional to the number of identical MLST profiles. Bold lines connect types that differ by one single locus. Halos indicate clusters. Colors indicate ethnic group; green: native Surinamese participants (n = 155); orange: native Dutch participants (n = 166); purple: Surinamese migrant participants (n = 64); and white: Dutch migrant participants (n = 2), other participants (n = 33) and participants with missing data (n = 6).

From the MLST profiles of these 426 samples, a minimum spanning tree was generated (*Figure 2*, *Figure 3*). Within this minimum spanning tree, identical samples or samples differing by one locus were grouped together. If these groups contained 25 or more samples, they were considered a cluster and this is indicated in the minimum spanning tree with a halo. A cluster supposedly represents one *C. trachomatis* strain type. In the minimum spanning tree generated from the 426 samples, we could identify four clusters (n = 53–105). Cluster 1 included 105 individuals and consisted mainly of genovar F samples (77%), but also of genovar D (19%) and J (4%). Cluster 2 (n = 76) and cluster 3 (n = 62) included solely genovar E samples. Cluster 4 (n = 53) consisted predominantly of genovar I (89%), but also of genovar J (11%). Among the residual samples (n = 130), all genovars were present, but genovars D (31%), G (19%), E (14%), K (12%) and J (10%) were most common.

In contrast to the genovar distribution, the distribution of samples in the MLST clusters was strongly associated with the city of sampling (*p*<0.001, *Table 1*; *Figure 2*). Overall, 60% of samples were from Amsterdam, but 88% of samples in cluster 2 were from Amsterdam, while 40% of samples in cluster 3 were from Amsterdam. No important deviations as expected from the overall sample distribution were found for cluster 1 (with 59% of the samples originating from Amsterdam), cluster 4 (with 49%), and the residual group (with 58%).

### Demographic characteristics of the clusters

Demographic characteristics were compared for the four large clusters and the residual group. Gender, age, and number of sexual partners in the past 12 months did not differ significantly between the clusters. However, significant differences in education (*p* = 0.003) and ethnic groups (*p*<0.001) were observed between the clusters (*Table S2*). The participants in cluster 2 had received more education, whereas participants in clusters 3 and 4 had received less education; these differences are largely due to the city of origin of the participants. Clusters 3 and 4 had more native Surinamese participants than the other clusters and cluster 2 had more native Dutch participants. Cluster 4 had more Surinamese migrant participants compared with the other clusters.

Because the distribution of *C. trachomatis* strains differed between the participants recruited in Amsterdam and Paramaribo, we also analyzed the data stratified by city of sampling (*Table S3*). In the analysis for the samples from Paramaribo, no significant differences were found between the clusters and any of the variables tested (gender, age, education, number of sexual partners, or ethnic group). For the Amsterdam samples, no significant differences were found between the clusters and gender, age, education, or number of sexual partners, but a very significant difference was found for ethnic group (*p*<0.001): 73% of samples in cluster 4 were from migrant Surinamese participants, while this group represented only 24% of the participants from Amsterdam (*Figure 3*, *Table S3*).

When we analyzed the data stratified for three main ethnic groups, no significant differences were found between the clusters and any of the variables tested for the native Surinamese, the native Dutch, or the Surinamese migrants (*Table S4*).

### Sexual mixing between the ethnic groups

We hypothesized that Surinamese migrants constituted a bridge population for the transmission of *C. trachomatis* between Suriname and the Netherlands. To investigate whether this pattern of transmission of *C. trachomatis* strains occurred, we investigated the degree of sexual mixing between the three main ethnic groups, i.e. native Surinamese participants (n = 155), Surinamese migrant participants (n = 64), and native Dutch participants (n = 166; *Figure 1*). In this analysis, we excluded the Dutch migrant participants (n = 2) and the other participants (n = 33), due to their low numbers and limited relevance in the model. In addition, 2 native Surinamese participants, 4 Surinamese migrant participants, and 16 native Dutch participants were excluded, as data regarding their sexual partners were missing or incomplete.

**Figure 4:**
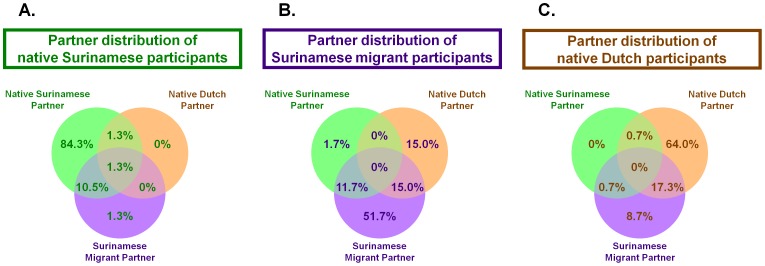
Venn diagrams of sexual mixing with native Surinamese partners (green circle), native Dutch partners (orange circle) and Surinamese migrant partners (purple circle) in the past 12 months. A. Native Surinamese participants (n = 153). B. Surinamese migrant participants (n = 60). C. Native Dutch participants (n = 150). Partner data were missing for 2 native Surinamese participants, 4 Surinamese migrant participants, and 16 native Dutch participants.

Among the Surinamese migrant participants, we found that 13% (n = 8/60) reported having had sex with a native Surinamese partner in the past 12 months, and 13% (n = 20/153) of the native Surinamese participants reported having had sex with a Surinamese migrant partner (*Figure 4*). Among Surinamese migrant participants, 30% (n = 18/60) reported having had sex with a native Dutch partner in the past 12 months, and 27% (n = 40/150) of the native Dutch participants reported having had sex with a Surinamese migrant partner. Among the native Surinamese participants we found that 3% (n = 4/153) reported having had sex with a native Dutch partner in the past 12 months, and 1% (n = 2/150) of the native Dutch participants reported having had sex with a native Surinamese partner.

**Figure 5:**
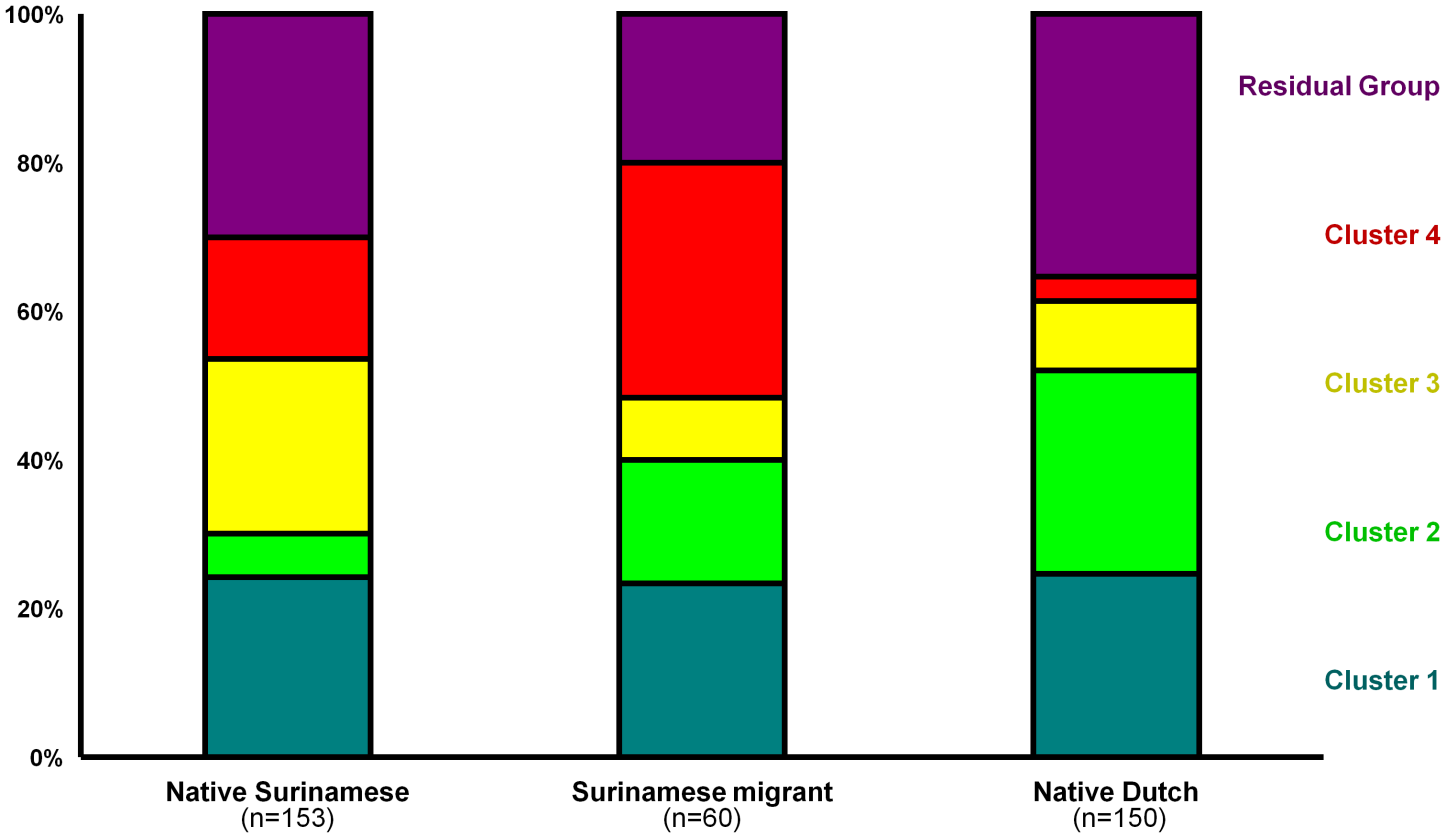
Distribution of the *Chlamydia trachomatis* clusters per ethnic group. Colors indicate the different clusters; dark green: cluster 1; light green: cluster 2; yellow: cluster 3; red: cluster 4; and purple: the residual group. These distribution of *C. trachomatis* strains differed significantly between the groups: native Surinamese participants – Surinamese migrant participants: p = 0.002; and Surinamese migrant participants – native Dutch participants: p<0.001.

Clear differences were found in the distribution of the clusters, including the residual group, between the three main ethnic groups: native Surinamese, Surinamese migrants and native Dutch (*Figure 5*). Compared with the native Surinamese participants, samples from the Surinamese migrant participants more often belonged to clusters 2 and 4, and less often to cluster 3 and the residual group (*p* = 0.002). Compared with the native Dutch participants, samples from the Surinamese migrant participants more often belonged to cluster 4 and less often to cluster 2 and the residual group (*p*<0.001). In addition, the distribution of the clusters found among the Surinamese migrants differed significantly from any intermediate state between the distribution found among the native Surinamese and native Dutch (*figure S1*).

The distribution of *C. trachomatis* strains found among the Surinamese migrant participants did not differ significantly between those who reported sex with a native Surinamese partner and those who did not (*p* = 0.43; *Table 2*). In addition, no significant differences were found in the distribution of *C. trachomatis* strains found among the native Surinamese participants who reported sex with a Surinamese migrant partner and those who did not (*p* = 0.13). The same was observed for the Surinamese migrant participants: no significant differences were found in *C. trachomatis* strain distribution between participants who reported and those who did not report sex with native Dutch partners (p = 0.66). Also among native Dutch participants no significant differences were found in *C. trachomatis* strain distribution between those who reported sex with Surinamese migrant partners and those who did not (p = 0.34).

**Table 2:** Sexual mixing with other ethnic groups among participants, by *Chlamydia trachomatis* cluster, Paramaribo, Suriname and Amsterdam, the Netherlands, 2008–10.

		Cluster 1	Cluster 2	Cluster 3	Cluster 4	Residual group	
		n (%)	n (%)	n (%)	n (%)	n (%)	*p*
*Native Surinamese participants (n = 153)*							
**Sex with Surinamese migrant partner:**	Yes	5 (14)	0 (0)	3 (8)	7 (28)	5 (11)	**0.13**
	No	32 (86)	9 (100)	33 (92)	18 (72)	41 (89)	
*Surinamese migrant participants (n = 60)*							
**Sex with native Surinamese partner:**	Yes	1 (7)	0 (0)	1 (20)	3 (16)	3 (25)	**0.43**
	No	13 (93)	10 (100)	4 (80)	16 (84)	9 (75)	
*Surinamese migrant participants (n = 60)*							
**Sex with native Dutch partner:**	Yes	4 (29)	5 (50)	1 (20)	5 (26)	3 (25)	**0.66**
	No	10 (71)	5 (50)	4 (80)	14 (74)	9 (75)	
*Native Dutch participants (n = 150)*							
**Sex with Surinamese migrant partner:**	Yes	10 (27)	10 (24)	7 (50)	1 (20)	12 (23)	**0.34**
	No	27 (73)	31 (76)	7 (50)	4 (80)	41 (77)	

## Discussion

In this study, we showed differences in the distribution of *C. trachomatis* strains between infected persons in Paramaribo and Amsterdam, and between Surinamese migrants and native Dutch participants within Amsterdam using the high-resolution MLST genotyping technique. These differences would have been largely obscured if conventional *ompA* typing had been used. For example, cluster 2 infections were more prevalent in Amsterdam and cluster 3 infections were more prevalent in Paramaribo, but both clusters were composed of MLST sequence types with an identical *ompA* type, genovar E, which is the most prevalent genovar type worldwide [13].

We hypothesized that the Surinamese migrant population residing in the Netherlands, who travel frequently between the two countries, acted as a bridge population for the transmission of *C. trachomatis* strains between inhabitants in Suriname and the Netherlands. We demonstrated that sexual mixing among Surinamese migrants occurred, both with native Surinamese and with native Dutch partners (13% and 30%). Sexual mixing between the native Dutch and native Surinamese groups occurred infrequently (1–3%). This indicates, based on reported sexual mixing, that Surinamese migrants potentially constituted a bridge population for STI between the Dutch and Surinamese populations. However, the distribution of *C. trachomatis* strains found in the Surinamese migrant population differed from both the native Surinamese and Dutch distributions, but was not an intermediate state between the native groups. This is most strikingly illustrated by the overrepresentation of Surinamese migrants in cluster 4, whereas cluster 4 strains are less prevalent among the native Surinamese population and rare in the native Dutch population. Therefore we conclude that limited transmission of *C. trachomatis* strains occurred between the native Surinamese and Surinamese migrant populations, and between the native Dutch and Surinamese migrant populations. In addition, when we examined the distribution of *C. trachomatis* strains among the participants that reported sexual mixing and those who did not, no significant differences could be found between the two in the distribution of *C. trachomatis* clusters. Although the power to show differences between these groups was limited, transmission patterns that could be expected were not observed. For example, among native Dutch participants who mix with Surinamese migrants more cluster 4 strains were expected compared to those who do not mix, as these strains were highly prevalent among the Surinamese migrant participants. However, just 5 Dutch participants were diagnosed with a cluster 4 strain and only 1 of them reported sex with Surinamese migrant partner past 12 months. Consequently, the high prevalence among the Surinamese migrants compared with the native Dutch cannot be explained by sexual mixing with native Surinamese.

In fact, the opposite may be true; both the high prevalence and the divergent distribution of *C. trachomatis* strains among the Surinamese migrants might be explained by the absence of effective sexual mixing with the native Dutch and Surinamese populations. When subpopulations do not mix effectively, infections cannot spread to other risk populations, and differences in prevalence are sustained [14], [15]. Similar patterns can be found among African-Americans where a higher prevalence in *C. trachomatis* infections is associated with socio-economic status and partnership structures [16], [17]. The differences in distribution of *C. trachomatis* strains can be explained assuming that the chlamydial populations in communities that do not mix are more susceptible to stochastic effects.

We were able to include a large number of participants from Suriname and the Netherlands, within a timespan of just over 2 years. Consequently we obtained an accurate representation of *C. trachomatis* strains circulating in both countries. Participants completed a questionnaire that was designed to study sexual networks, so detailed epidemiological data were available and these could be linked to high-resolution typing results of the pathogen. Together, these factors enabled us to study the determinants of the specific groups for the transmission of *C. trachomatis*. Although the data of the participants' ethnic origin was precise (based on their own assessment), the data of their sexual partners was limited to ‘perceived ethnicity’. Therefore the amount of sexual mixing between the groups may be overreported, especially for native Dutch and Surinamese migrant partners. The native Dutch partners were defined as a partner of a perceived Caucasian ethnicity living in the Netherlands. Likely, this group encompasses more than only the native Dutch, as Caucasian minorities also reside in Amsterdam. More biased are the Surinamese migrant partners, as they were defined as a partner of a perceived Creole, Hindustani, Javanese, Chinese, Maroon, Amerindian, or ‘mixed race’ ethnicity living in the Netherlands. Whereas these ethnic groups are specific for native and migrant Suriname individuals, some of these ethnicities are not distinguishable for native Dutch participants, especially the ‘mixed race’ ethnicity. In addition, native Dutch individuals might be unaware of Surinamese ethnic groups and therefore underreport them.

Despite the limitations, these data provide a good view of the *C. trachomatis* strains circulating in Suriname and the Netherlands, and the limited transmission of these strains between the two countries. Although our data do not seem to justify the need for joint campaigns to reduce the transmission of *C. trachomatis* strains between both countries, intensified preventive campaigns to decrease the *C. trachomatis* burden are required, both in Suriname and in the Netherlands. Moreover, informing travelers about the risks of unprotected sex abroad is still relevant. In addition, the high prevalence of *C. trachomatis* within the Surinamese migrant population justifies further studies into risk factors and transmission networks of *C. trachomatis* within this group. This will enable more effective tailored prevention programs to reduce the *C. trachomatis* burden.

## Supporting Information

Figure S1 **Test for intermediacy.** Depicted are the p-values of the *C. trachomatis* strain distribution found among the Surinamese migrants compared with the distributions of the hypothetical intermediate states, using Pearson's χ^2^ tests. These intermediate states ranged from 100% identical to the native Surinamese distribution to 100% identical to the native Dutch distribution. None of these intermediate states had a p-value of ≥0.05, therefore the distribution of *C. trachomatis* strains found among the Surinamese migrants was not an intermediate state between the distributions found among the native Surinamese and native Dutch populations.
(TIF)

Table S1 **MLST-data of the 426 samples collected in Paramaribo, Suriname and Amsterdam, the Netherlands, 2008–10.** Coding is according to the *Chlamydia trachomatis* MLST database (mlstdb.bmc.uu.se). Samples S1001 to S1170 were collected in Paramaribo, and samples 3003 to 3329 were collected in Amsterdam.
(DOC)

Table S2 **Characteristics of** ***Chlamydia trachomatis*** **-positive participants, by** ***C. trachomatis*** **cluster, from Paramaribo, Suriname and Amsterdam, the Netherlands, 2008–10.**
(DOC)

Table S3 **Characteristics of** ***Chlamydia trachomatis*** **-positive participants, by** ***C. trachomatis*** **cluster.** From Paramaribo, Suriname, 2008–10 (A), and from Amsterdam, the Netherlands, 2009–10 (B).
(DOCX)

Table S4 Characteristics of *Chlamydia trachomatis*-positive native Surinamese participants, by *C. trachomatis* cluster, 2008–10 (A). Characteristics of *Chlamydia trachomatis*-positive native Dutch participants, by *C. trachomatis* cluster, 2009–10 (B). Characteristics of *Chlamydia trachomatis*-positive Surinamese migrant participants, by *C. trachomatis* cluster, 2009–10 (C).
(DOCX)
