# Supplementary figures and images for: The Role of Surinamese Migrants in the Transmission of Chlamydia trachomatis between Paramaribo, Suriname and Amsterdam, The Netherlands
Source: PLoS One. 2013 Nov 13;8(11):e77977. doi: 10.1371/journal.pone.0077977 (PMC3827209; doi:10.1371/journal.pone.0077977)

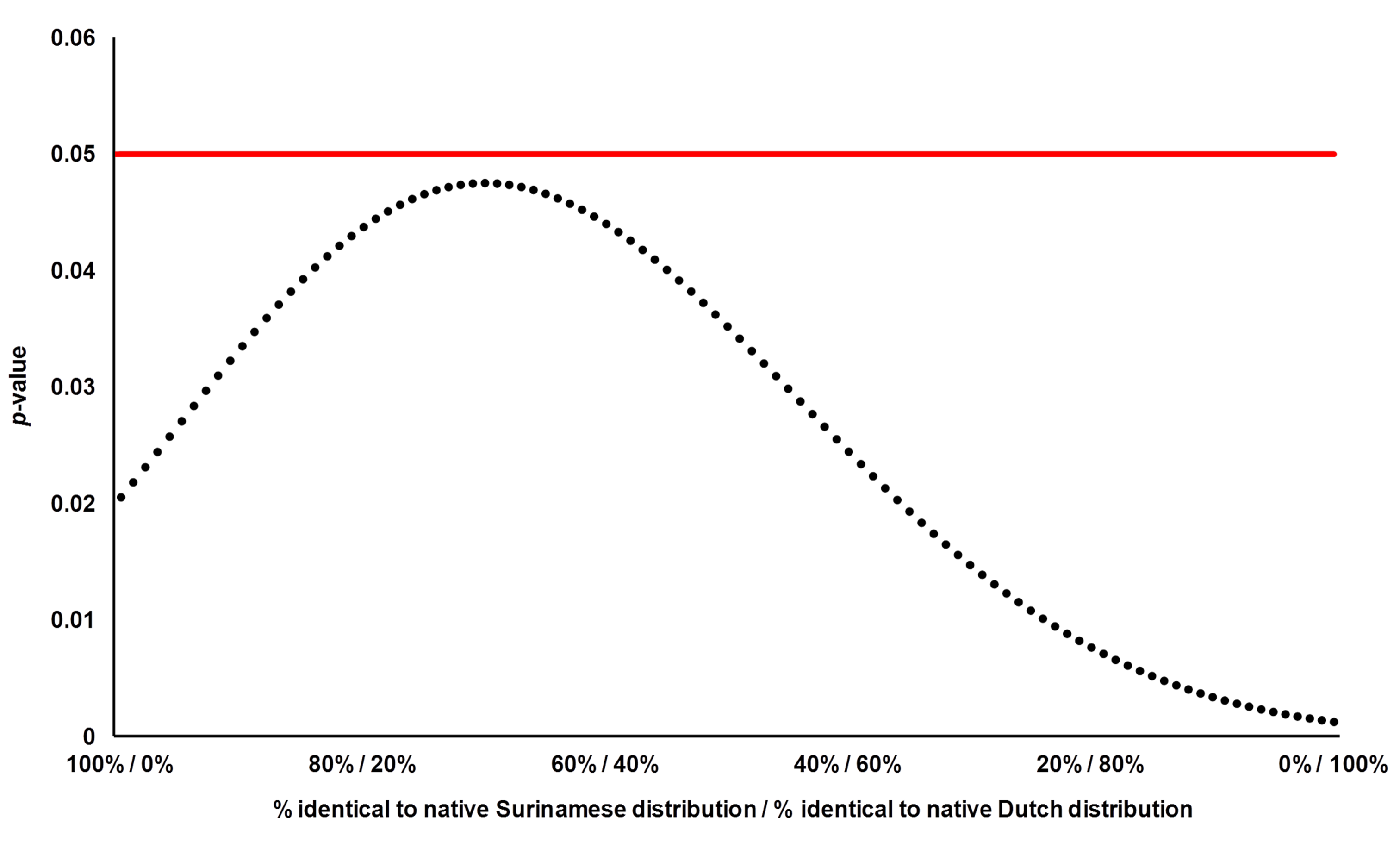

Supplement: Figure S1 — Test for intermediacy. Depicted are the p-values of the C. trachomatis strain distribution found among the Surinamese migrants compared with the distributions of the hypothetical intermediate states, using Pearson's χ2 tests. These intermediate states ranged from 100% identical to the native Surinamese distribution to 100% identical to the native Dutch distribution. None of these intermediate states had a p-value of ≥0.05, therefore the distribution of C. trachomatis strains found among the Surinamese migrants was not an intermediate state between the distributions found among the native Surinamese and native Dutch populations. (TIF) [file pone.0077977.s001.tif]
