# Supplementary material for: The Role of Surinamese Migrants in the Transmission of Chlamydia trachomatis between Paramaribo, Suriname and Amsterdam, The Netherlands
Source: PLoS One. 2013 Nov 13;8(11):e77977. doi: 10.1371/journal.pone.0077977 (PMC3827209; doi:10.1371/journal.pone.0077977)
Supplement: Table S1 — MLST-data of the 426 samples collected in Paramaribo, Suriname and Amsterdam, the Netherlands, 2008–10. Coding is according to the Chlamydia trachomatis MLST database (mlstdb.bmc.uu.se). Samples S1001 to S1170 were collected in Paramaribo, and samples 3003 to 3329 were collected in Amsterdam. (DOC) [file pone.0077977.s002.doc]

*Table S1. MLST-data of the 426 samples collected in Paramaribo, Suriname and Amsterdam, the Netherlands, 2008-10. Coding is according to the Chlamydia trachomatis MLST database (mlstdb.bmc.uu.se). Samples S1001 to S1170 were collected in Paramaribo, and samples 3003 to 3329 were collected in Amsterdam.*

| **Sample ID** | **MLST-6 ST** | **genovar** | ***ompA* (CT681)** | ***hctB* (CT046)** | **CT058** | **CT144** | **CT172** | ***pbpB* (CT682)** |
| --- | --- | --- | --- | --- | --- | --- | --- | --- |
| **S1001** | 3 | E | 6 | 1 | 2 | 6 | 2 | 2 |
| **S1002** | 269 | I | 36 | 10 | 5 | 12 | 2 | 18 |
| **S1003** | 351 | I | 36 | 79 | 5 | 12 | 27 | 18 |
| **S1004** | 248 | D | 1 | 5 | 28 | 7 | 1 | 4 |
| **S1005** | 223 | E | 6 | 1 | 5 | 12 | 2 | 2 |
| **S1006** | 3 | E | 6 | 1 | 2 | 6 | 2 | 2 |
| **S1007** | 274 | I | 36 | 10 | 5 | 12 | 26 | 18 |
| **S1008** | 228 | E | 6 | 5 | 2 | 22 | 2 | 2 |
| **S1009** | 3 | E | 6 | 1 | 2 | 6 | 2 | 2 |
| **S1010** | 136a | J | 20 | 10 | 5 | 12 | 3 | 18 |
| **S1011** | 268a | J | 20 | 10 | 5 | 12 | 1 | 18 |
| **S1012** | 98a | F | 24 | 37 | 15 | 7 | 1 | 4 |
| **S1013** | 136a | J | 20 | 10 | 5 | 12 | 3 | 18 |
| **S1014** | 280 | B | 30 | 10 | 7 | 1 | 26 | 54 |
| **S1015** | 273 | I | 36 | 10 | 5 | 12 | 25 | 18 |
| **S1016** | 347 | G | 8 | 76 | 8 | 22 | 28 | 6 |
| **S1017** | 12d | F | 24 | 5 | 19 | 7 | 1 | 4 |
| **S1018** | 3 | E | 6 | 1 | 2 | 6 | 2 | 2 |
| **S1019** | 3 | E | 6 | 1 | 2 | 6 | 2 | 2 |
| **S1020** | 153 | E | 6 | 35 | 19 | 7 | 2 | 1 |
| **S1021** | 314 | E | 6 | 37 | 15 | 7 | 1 | 2 |
| **S1022** | 3 | E | 6 | 1 | 2 | 6 | 2 | 2 |
| **S1023** | 12d | F | 24 | 5 | 19 | 7 | 1 | 4 |
| **S1024** | 153 | E | 6 | 35 | 19 | 7 | 2 | 1 |
| **S1025** | 276 | I | 36 | 10 | 5 | 12 | 27 | 18 |
| **S1026** | 314 | E | 6 | 37 | 15 | 7 | 1 | 2 |
| **S1027** | 62b | F | 24 | 5 | 19 | 7 | 3 | 4 |
| **S1028** | 3 | E | 6 | 1 | 2 | 6 | 2 | 2 |
| **S1029** | 34b | K | 12 | 10 | 8 | 1 | 4 | 8 |
| **S1030** | 268a | J | 20 | 10 | 5 | 12 | 1 | 18 |
| **S1031** | 229 | D | 1 | 5 | 2 | 22 | 2 | 37 |
| **S1032** | 78 | D | 1 | 7 | 19 | 18 | 2 | 34 |
| **S1033** | 77b | D | 31 | 5 | 19 | 7 | 2 | 37 |
| **S1034** | 91a | F | 24 | 5 | 19 | 5 | 2 | 4 |
| **S1035** | 248 | D | 1 | 5 | 28 | 7 | 1 | 4 |
| **S1036** | 248 | D | 1 | 5 | 28 | 7 | 1 | 4 |
| **S1037** | 248 | D | 1 | 5 | 28 | 7 | 1 | 4 |
| **S1038** | 62b | F | 24 | 5 | 19 | 7 | 3 | 4 |
| **S1039** | 35 | D | 2 | 10 | 8 | 1 | 4 | 17 |
| **S1040** | 78 | D | 1 | 7 | 19 | 18 | 2 | 34 |
| **S1041** | 322 | I | 36 | 53 | 5 | 12 | 25 | 18 |
| **S1042** | 265 | G | 10 | 10 | 4 | 1 | 3 | 7 |
| **S1043** | 100b | I | 36 | 10 | 5 | 12 | 7 | 18 |
| **S1044** | 330 | E | 6 | 60 | 2 | 6 | 2 | 2 |
| **S1045** | 12d | F | 24 | 5 | 19 | 7 | 1 | 4 |
| **S1046** | 12d | F | 24 | 5 | 19 | 7 | 1 | 4 |
| **S1047** | 91a | F | 24 | 5 | 19 | 5 | 2 | 4 |
| **S1048** | 249 | E | 6 | 5 | 43 | 7 | 1 | 56 |
| **S1049** | 295 | J | 20 | 12 | 5 | 2 | 2 | 18 |
| **S1050** | 329 | E | 6 | 59 | 2 | 6 | 2 | 2 |
| **S1051** | 100b | I | 36 | 10 | 5 | 12 | 7 | 18 |
| **S1052** | 233 | J | 38 | 5 | 19 | 5 | 1 | 4 |
| **S1053** | 350 | I | 36 | 79 | 5 | 12 | 26 | 18 |
| **S1054** | 260 | G | 8 | 8 | 8 | 22 | 3 | 6 |
| **S1055** | 101 | I | 36 | 38 | 5 | 12 | 7 | 18 |
| **S1056** | 77b | D | 31 | 5 | 19 | 7 | 2 | 37 |
| **S1057** | 293 | J | 20 | 11 | 6 | 22 | 4 | 8 |
| **S1058** | 285 | G | 8 | 10 | 8 | 22 | 3 | 6 |
| **S1059** | 230 | D | 31 | 5 | 5 | 7 | 2 | 37 |
| **S1060** | 101 | I | 36 | 38 | 5 | 12 | 7 | 18 |
| **S1061** | 284 | K | 12 | 10 | 8 | 12 | 3 | 8 |
| **S1062** | 249 | E | 6 | 5 | 43 | 7 | 1 | 56 |
| **S1063** | 100b | I | 36 | 10 | 5 | 12 | 7 | 18 |
| **S1064** | 248 | D | 1 | 5 | 28 | 7 | 1 | 4 |
| **S1065** | 12d | F | 24 | 5 | 19 | 7 | 1 | 4 |
| **S1066** | 3 | E | 6 | 1 | 2 | 6 | 2 | 2 |
| **S1067** | 3 | E | 6 | 1 | 2 | 6 | 2 | 2 |
| **S1068** | 273 | I | 36 | 10 | 5 | 12 | 25 | 18 |
| **S1069** | 12d | F | 24 | 5 | 19 | 7 | 1 | 4 |
| **S1070** | 34a | D | 2 | 10 | 8 | 1 | 4 | 8 |
| **S1071** | 87 | E | 6 | 35 | 2 | 6 | 2 | 2 |
| **S1072** | 77b | D | 31 | 5 | 19 | 7 | 2 | 37 |
| **S1073** | 12d | F | 24 | 5 | 19 | 7 | 1 | 4 |
| **S1074** | 339 | E | 6 | 68 | 19 | 7 | 3 | 2 |
| **S1075** | 3 | E | 6 | 1 | 2 | 6 | 2 | 2 |
| **S1076** | 34b | K | 12 | 10 | 8 | 1 | 4 | 8 |
| **S1077** | 3 | E | 6 | 1 | 2 | 6 | 2 | 2 |
| **S1078** | 12d | F | 24 | 5 | 19 | 7 | 1 | 4 |
| **S1079** | 228 | E | 6 | 5 | 2 | 22 | 2 | 2 |
| **S1080** | 12d | F | 24 | 5 | 19 | 7 | 1 | 4 |
| **S1081** | 291 | D | 2 | 10 | 47 | 1 | 4 | 17 |
| **S1082** | 56a | E | 6 | 1 | 19 | 7 | 2 | 1 |
| **S1083** | 3 | E | 6 | 1 | 2 | 6 | 2 | 2 |
| **S1084** | 276 | I | 36 | 10 | 5 | 12 | 27 | 18 |
| **S1085** | 12d | F | 24 | 5 | 19 | 7 | 1 | 4 |
| **S1086** | 77b | D | 31 | 5 | 19 | 7 | 2 | 37 |
| **S1087** | 3 | E | 6 | 1 | 2 | 6 | 2 | 2 |
| **S1088** | 77b | D | 31 | 5 | 19 | 7 | 2 | 37 |
| **S1089** | 135a | J | 20 | 10 | 5 | 12 | 4 | 18 |
| **S1090** | 59 | E | 6 | 7 | 19 | 7 | 2 | 1 |
| **S1091** | 3 | E | 6 | 1 | 2 | 6 | 2 | 2 |
| **S1092** | 74 | B | 30 | 8 | 8 | 1 | 7 | 18 |
| **S1093** | 281b | J | 63 | 10 | 8 | 1 | 4 | 18 |
| **S1094** | 335 | J | 38 | 64 | 55 | 5 | 18 | 10 |
| **S1095** | 322 | I | 36 | 53 | 5 | 12 | 25 | 18 |
| **S1096** | 77b | D | 31 | 5 | 19 | 7 | 2 | 37 |
| **S1097** | 314 | E | 6 | 37 | 15 | 7 | 1 | 2 |
| **S1098** | 35 | D | 2 | 10 | 8 | 1 | 4 | 17 |
| **S1099** | 3 | E | 6 | 1 | 2 | 6 | 2 | 2 |
| **S1100** | 35 | D | 2 | 10 | 8 | 1 | 4 | 17 |
| **S1101** | 351 | I | 36 | 79 | 5 | 12 | 27 | 18 |
| **S1102** | 62b | F | 24 | 5 | 19 | 7 | 3 | 4 |
| **S1103** | 3 | E | 6 | 1 | 2 | 6 | 2 | 2 |
| **S1104** | 109 | D | 1 | 5 | 20 | 5 | 2 | 34 |
| **S1105** | 228 | E | 6 | 5 | 2 | 22 | 2 | 2 |
| **S1106** | 3 | E | 6 | 1 | 2 | 6 | 2 | 2 |
| **S1107** | 172 | E | 6 | 1 | 2 | 7 | 2 | 2 |
| **S1108** | 109 | D | 1 | 5 | 20 | 5 | 2 | 34 |
| **S1109** | 12d | F | 24 | 5 | 19 | 7 | 1 | 4 |
| **S1110** | 62b | F | 24 | 5 | 19 | 7 | 3 | 4 |
| **S1111** | 3 | E | 6 | 1 | 2 | 6 | 2 | 2 |
| **S1112** | 276 | I | 36 | 10 | 5 | 12 | 27 | 18 |
| **S1113** | 12d | F | 24 | 5 | 19 | 7 | 1 | 4 |
| **S1114** | 3 | E | 6 | 1 | 2 | 6 | 2 | 2 |
| **S1115** | 12a | D | 1 | 5 | 19 | 7 | 1 | 4 |
| **S1116** | 100b | I | 36 | 10 | 5 | 12 | 7 | 18 |
| **S1117** | 56a | E | 6 | 1 | 19 | 7 | 2 | 1 |
| **S1118** | 276 | I | 36 | 10 | 5 | 12 | 27 | 18 |
| **S1119** | 12d | F | 24 | 5 | 19 | 7 | 1 | 4 |
| **S1120** | 91a | F | 24 | 5 | 19 | 5 | 2 | 4 |
| **S1121** | 91a | F | 24 | 5 | 19 | 5 | 2 | 4 |
| **S1122** | 13a | D | 2 | 5 | 19 | 15 | 1 | 4 |
| **S1123** | 101 | I | 36 | 38 | 5 | 12 | 7 | 18 |
| **S1124** | 248 | D | 1 | 5 | 28 | 7 | 1 | 4 |
| **S1125** | 292 | G | 9 | 10 | 49 | 10 | 4 | 6 |
| **S1126** | 12d | F | 24 | 5 | 19 | 7 | 1 | 4 |
| **S1127** | 282 | H | 18 | 10 | 8 | 6 | 4 | 8 |
| **S1128** | 77b | D | 31 | 5 | 19 | 7 | 2 | 37 |
| **S1129** | 291 | D | 2 | 10 | 47 | 1 | 4 | 17 |
| **S1130** | 239 | E | 6 | 5 | 19 | 7 | 20 | 2 |
| **S1131** | 330 | E | 6 | 60 | 2 | 6 | 2 | 2 |
| **S1132** | 34b | K | 12 | 10 | 8 | 1 | 4 | 8 |
| **S1133** | 3 | E | 6 | 1 | 2 | 6 | 2 | 2 |
| **S1134** | 13a | D | 2 | 5 | 19 | 15 | 1 | 4 |
| **S1135** | 3 | E | 6 | 1 | 2 | 6 | 2 | 2 |
| **S1136** | 241 | D | 1 | 5 | 19 | 33 | 2 | 37 |
| **S1137** | 12d | F | 24 | 5 | 19 | 7 | 1 | 4 |
| **S1138** | 3 | E | 6 | 1 | 2 | 6 | 2 | 2 |
| **S1139** | 100b | I | 36 | 10 | 5 | 12 | 7 | 18 |
| **S1140** | 330 | E | 6 | 60 | 2 | 6 | 2 | 2 |
| **S1141** | 3 | E | 6 | 1 | 2 | 6 | 2 | 2 |
| **S1142** | 3 | E | 6 | 1 | 2 | 6 | 2 | 2 |
| **S1143** | 56a | E | 6 | 1 | 19 | 7 | 2 | 1 |
| **S1144** | 319 | E | 6 | 49 | 2 | 6 | 2 | 2 |
| **S1145** | 3 | E | 6 | 1 | 2 | 6 | 2 | 2 |
| **S1146** | 12d | F | 24 | 5 | 19 | 7 | 1 | 4 |
| **S1147** | 12d | F | 24 | 5 | 19 | 7 | 1 | 4 |
| **S1148** | 157 | E | 6 | 5 | 19 | 7 | 1 | 1 |
| **S1149** | 56a | E | 6 | 1 | 19 | 7 | 2 | 1 |
| **S1150** | 268b | I | 36 | 10 | 5 | 12 | 1 | 18 |
| **S1151** | 3 | E | 6 | 1 | 2 | 6 | 2 | 2 |
| **S1152** | 62b | F | 24 | 5 | 19 | 7 | 3 | 4 |
| **S1153** | 248 | D | 1 | 5 | 28 | 7 | 1 | 4 |
| **S1154** | 211 | B | 55 | 10 | 8 | 1 | 1 | 18 |
| **S1155** | 3 | E | 6 | 1 | 2 | 6 | 2 | 2 |
| **S1156** | 137 | G | 8 | 10 | 8 | 22 | 4 | 6 |
| **S1157** | 34b | K | 12 | 10 | 8 | 1 | 4 | 8 |
| **S1158** | 12d | F | 24 | 5 | 19 | 7 | 1 | 4 |
| **S1159** | 260 | G | 8 | 8 | 8 | 22 | 3 | 6 |
| **S1160** | 336 | F | 24 | 65 | 19 | 7 | 1 | 4 |
| **S1161** | 91a | F | 24 | 5 | 19 | 5 | 2 | 4 |
| **S1162** | 3 | E | 6 | 1 | 2 | 6 | 2 | 2 |
| **S1163** | 87 | E | 6 | 35 | 2 | 6 | 2 | 2 |
| **S1164** | 260 | G | 8 | 8 | 8 | 22 | 3 | 6 |
| **S1165** | 229 | D | 1 | 5 | 2 | 22 | 2 | 37 |
| **S1166** | 91a | F | 24 | 5 | 19 | 5 | 2 | 4 |
| **S1167** | 62b | F | 24 | 5 | 19 | 7 | 3 | 4 |
| **S1168** | 165 | H | 35 | 12 | 5 | 1 | 9 | 8 |
| **S1169** | 261 | G | 8 | 8 | 8 | 35 | 3 | 6 |
| **S1170** | 62b | F | 24 | 5 | 19 | 7 | 3 | 4 |
| **3003** | 171 | E | 6 | 49 | 19 | 7 | 2 | 1 |
| **3004** | 12d | F | 24 | 5 | 19 | 7 | 1 | 4 |
| **3005** | 12d | F | 24 | 5 | 19 | 7 | 1 | 4 |
| **3006** | 234 | D | 31 | 5 | 19 | 6 | 2 | 37 |
| **3007** | 297 | F | 24 | 13 | 19 | 7 | 1 | 4 |
| **3008** | 87 | E | 6 | 35 | 2 | 6 | 2 | 2 |
| **3009** | 56a | E | 6 | 1 | 19 | 7 | 2 | 1 |
| **3010** | 320 | E | 6 | 49 | 19 | 7 | 23 | 1 |
| **3011** | 153 | E | 6 | 35 | 19 | 7 | 2 | 1 |
| **3012** | 25b | I | 19 | 10 | 4 | 1 | 7 | 6 |
| **3013** | 59 | E | 6 | 7 | 19 | 7 | 2 | 1 |
| **3014** | 135b | I | 36 | 10 | 5 | 12 | 4 | 18 |
| **3015** | 35 | D | 2 | 10 | 8 | 1 | 4 | 17 |
| **3016** | 30 | K | 12 | 10 | 7 | 1 | 3 | 8 |
| **3017** | 13a | D | 2 | 5 | 19 | 15 | 1 | 4 |
| **3018** | 90 | F | 24 | 5 | 19 | 1 | 1 | 4 |
| **3020** | 235 | D | 1 | 5 | 19 | 7 | 1 | 59 |
| **3021** | 16 | E | 6 | 7 | 19 | 14 | 2 | 1 |
| **3022** | 3 | E | 6 | 1 | 2 | 6 | 2 | 2 |
| **3023** | 56a | E | 6 | 1 | 19 | 7 | 2 | 1 |
| **3024** | 297 | F | 24 | 13 | 19 | 7 | 1 | 4 |
| **3025** | 153 | E | 6 | 35 | 19 | 7 | 2 | 1 |
| **3026** | 253 | J | 20 | 7 | 19 | 7 | 2 | 18 |
| **3027** | 3 | E | 6 | 1 | 2 | 6 | 2 | 2 |
| **3028** | 32 | K | 12 | 10 | 7 | 1 | 4 | 8 |
| **3029** | 3 | E | 6 | 1 | 2 | 6 | 2 | 2 |
| **3030** | 12d | F | 24 | 5 | 19 | 7 | 1 | 4 |
| **3032** | 12d | F | 24 | 5 | 19 | 7 | 1 | 4 |
| **3033** | 56a | E | 6 | 1 | 19 | 7 | 2 | 1 |
| **3034** | 3 | E | 6 | 1 | 2 | 6 | 2 | 2 |
| **3036** | 3 | E | 6 | 1 | 2 | 6 | 2 | 2 |
| **3037** | 56a | E | 6 | 1 | 19 | 7 | 2 | 1 |
| **3039** | 233 | J | 38 | 5 | 19 | 5 | 1 | 4 |
| **3041** | 130a | D | 2 | 10 | 4 | 1 | 3 | 5 |
| **3043** | 56a | E | 6 | 1 | 19 | 7 | 2 | 1 |
| **3044** | 128a | G | 8 | 10 | 8 | 1 | 4 | 5 |
| **3045** | 279 | I | 37 | 10 | 7 | 1 | 4 | 5 |
| **3046** | 12a | D | 1 | 5 | 19 | 7 | 1 | 4 |
| **3049** | 16 | E | 6 | 7 | 19 | 14 | 2 | 1 |
| **3050** | 275 | G | 8 | 10 | 5 | 12 | 27 | 5 |
| **3051** | 30 | K | 12 | 10 | 7 | 1 | 3 | 8 |
| **3052** | 12d | F | 24 | 5 | 19 | 7 | 1 | 4 |
| **3053** | 148 | F | 24 | 5 | 19 | 7 | 2 | 4 |
| **3054** | 290 | I | 37 | 10 | 46 | 1 | 7 | 5 |
| **3055** | 12d | F | 24 | 5 | 19 | 7 | 1 | 4 |
| **3056** | 277 | G | 8 | 10 | 6 | 1 | 3 | 6 |
| **3057** | 59 | E | 6 | 7 | 19 | 7 | 2 | 1 |
| **3058** | 59 | E | 6 | 7 | 19 | 7 | 2 | 1 |
| **3059** | 56c | E | 61 | 1 | 19 | 7 | 2 | 1 |
| **3060** | 12d | F | 24 | 5 | 19 | 7 | 1 | 4 |
| **3061** | 237 | E | 6 | 5 | 19 | 7 | 2 | 2 |
| **3062** | 12d | F | 24 | 5 | 19 | 7 | 1 | 4 |
| **3063** | 59 | E | 6 | 7 | 19 | 7 | 2 | 1 |
| **3064** | 128a | G | 8 | 10 | 8 | 1 | 4 | 5 |
| **3065** | 12d | F | 24 | 5 | 19 | 7 | 1 | 4 |
| **3066** | 12e | J | 38 | 5 | 19 | 7 | 1 | 4 |
| **3067** | 12d | F | 24 | 5 | 19 | 7 | 1 | 4 |
| **3069** | 287 | G | 8 | 10 | 8 | 22 | 7 | 6 |
| **3070** | 69 | E | 6 | 5 | 19 | 6 | 2 | 2 |
| **3071** | 56a | E | 6 | 1 | 19 | 7 | 2 | 1 |
| **3072** | 69 | E | 6 | 5 | 19 | 6 | 2 | 2 |
| **3073** | 148 | F | 24 | 5 | 19 | 7 | 2 | 4 |
| **3075** | 59 | E | 6 | 7 | 19 | 7 | 2 | 1 |
| **3076** | 56a | E | 6 | 1 | 19 | 7 | 2 | 1 |
| **3077** | 323 | E | 6 | 54 | 19 | 7 | 2 | 1 |
| **3078** | 3 | E | 6 | 1 | 2 | 6 | 2 | 2 |
| **3079** | 148 | F | 24 | 5 | 19 | 7 | 2 | 4 |
| **3080** | 165 | H | 35 | 12 | 5 | 1 | 9 | 8 |
| **3081** | 59 | E | 6 | 7 | 19 | 7 | 2 | 1 |
| **3082** | 135b | I | 36 | 10 | 5 | 12 | 4 | 18 |
| **3083** | 12d | F | 24 | 5 | 19 | 7 | 1 | 4 |
| **3084** | 56a | E | 6 | 1 | 19 | 7 | 2 | 1 |
| **3085** | 232 | J | 20 | 5 | 19 | 1 | 4 | 18 |
| **3088** | 288 | G | 8 | 10 | 8 | 22 | 7 | 57 |
| **3089** | 12d | F | 24 | 5 | 19 | 7 | 1 | 4 |
| **3090** | 355 | D | 2 | 83 | 4 | 1 | 4 | 17 |
| **3091** | 3 | E | 6 | 1 | 2 | 6 | 2 | 2 |
| **3092** | 25b | I | 19 | 10 | 4 | 1 | 7 | 6 |
| **3093** | 319 | E | 6 | 49 | 2 | 6 | 2 | 2 |
| **3094** | 12d | F | 24 | 5 | 19 | 7 | 1 | 4 |
| **3095** | 341 | E | 6 | 70 | 57 | 7 | 2 | 1 |
| **3096** | 263 | E | 6 | 9 | 15 | 6 | 2 | 2 |
| **3097** | 227 | E | 6 | 5 | 2 | 12 | 22 | 56 |
| **3098** | 3 | E | 6 | 1 | 2 | 6 | 2 | 2 |
| **3099** | 135b | I | 36 | 10 | 5 | 12 | 4 | 18 |
| **3100** | 12a | D | 1 | 5 | 19 | 7 | 1 | 4 |
| **3101** | 337 | F | 24 | 66 | 19 | 7 | 1 | 4 |
| **3102** | 12d | F | 24 | 5 | 19 | 7 | 1 | 4 |
| **3103** | 12d | F | 24 | 5 | 19 | 7 | 1 | 4 |
| **3104** | 276 | I | 36 | 10 | 5 | 12 | 27 | 18 |
| **3105** | 153 | E | 6 | 35 | 19 | 7 | 2 | 1 |
| **3106** | 59 | E | 6 | 7 | 19 | 7 | 2 | 1 |
| **3108** | 337 | F | 24 | 66 | 19 | 7 | 1 | 4 |
| **3109** | 102 | I | 37 | 10 | 7 | 1 | 7 | 5 |
| **3110** | 267 | J | 20 | 10 | 5 | 1 | 15 | 18 |
| **3111** | 337 | F | 24 | 66 | 19 | 7 | 1 | 4 |
| **3112** | 325 | E | 6 | 56 | 2 | 6 | 2 | 2 |
| **3113** | 59 | E | 6 | 7 | 19 | 7 | 2 | 1 |
| **3115** | 16 | E | 6 | 7 | 19 | 14 | 2 | 1 |
| **3117** | 12d | F | 24 | 5 | 19 | 7 | 1 | 4 |
| **3118** | 317 | I | 36 | 38 | 5 | 32 | 27 | 18 |
| **3119** | 288 | G | 8 | 10 | 8 | 22 | 7 | 57 |
| **3120** | 332 | F | 24 | 62 | 19 | 7 | 1 | 4 |
| **3122** | 274 | I | 36 | 10 | 5 | 12 | 26 | 18 |
| **3123** | 316 | I | 36 | 38 | 5 | 32 | 7 | 18 |
| **3124** | 276 | I | 36 | 10 | 5 | 12 | 27 | 18 |
| **3125** | 153 | E | 6 | 35 | 19 | 7 | 2 | 1 |
| **3126** | 56a | E | 6 | 1 | 19 | 7 | 2 | 1 |
| **3127** | 35 | D | 2 | 10 | 8 | 1 | 4 | 17 |
| **3128** | 12d | F | 24 | 5 | 19 | 7 | 1 | 4 |
| **3130** | 251 | F | 24 | 5 | 56 | 7 | 1 | 4 |
| **3131** | 154 | E | 6 | 48 | 19 | 7 | 2 | 1 |
| **3133** | 59 | E | 6 | 7 | 19 | 7 | 2 | 1 |
| **3134** | 313 | E | 6 | 35 | 54 | 7 | 2 | 1 |
| **3135** | 160 | E | 6 | 10 | 2 | 1 | 2 | 2 |
| **3138** | 32 | K | 12 | 10 | 7 | 1 | 4 | 8 |
| **3139** | 56a | E | 6 | 1 | 19 | 7 | 2 | 1 |
| **3141** | 222 | E | 6 | 1 | 5 | 6 | 2 | 2 |
| **3143** | 100b | I | 36 | 10 | 5 | 12 | 7 | 18 |
| **3145** | 16 | E | 6 | 7 | 19 | 14 | 2 | 1 |
| **3146** | 298 | D | 1 | 13 | 19 | 7 | 3 | 10 |
| **3147** | 56a | E | 6 | 1 | 19 | 7 | 2 | 1 |
| **3148** | 59 | E | 6 | 7 | 19 | 7 | 2 | 1 |
| **3150** | 259 | B | 30 | 8 | 8 | 1 | 4 | 18 |
| **3152** | 254 | E | 6 | 7 | 50 | 7 | 21 | 1 |
| **3153** | 12d | F | 24 | 5 | 19 | 7 | 1 | 4 |
| **3155** | 16 | E | 6 | 7 | 19 | 14 | 2 | 1 |
| **3156** | 138 | B | 30 | 11 | 7 | 12 | 3 | 18 |
| **3157** | 27 | G | 9 | 10 | 6 | 10 | 1 | 6 |
| **3159** | 90 | F | 24 | 5 | 19 | 1 | 1 | 4 |
| **3160** | 56a | E | 6 | 1 | 19 | 7 | 2 | 1 |
| **3161** | 331 | E | 6 | 61 | 19 | 7 | 2 | 1 |
| **3162** | 288 | G | 8 | 10 | 8 | 22 | 7 | 57 |
| **3163** | 77b | D | 31 | 5 | 19 | 7 | 2 | 37 |
| **3164** | 172 | E | 6 | 1 | 2 | 7 | 2 | 2 |
| **3165** | 328 | E | 6 | 58 | 2 | 6 | 2 | 2 |
| **3166** | 135b | I | 36 | 10 | 5 | 12 | 4 | 18 |
| **3167** | 272 | I | 36 | 10 | 5 | 12 | 24 | 18 |
| **3168** | 250 | F | 24 | 5 | 51 | 7 | 3 | 4 |
| **3169** | 337 | F | 24 | 66 | 19 | 7 | 1 | 4 |
| **3170** | 3 | E | 6 | 1 | 2 | 6 | 2 | 2 |
| **3172** | 56a | E | 6 | 1 | 19 | 7 | 2 | 1 |
| **3173** | 3 | E | 6 | 1 | 2 | 6 | 2 | 2 |
| **3175** | 59 | E | 6 | 7 | 19 | 7 | 2 | 1 |
| **3176** | 69 | E | 6 | 5 | 19 | 6 | 2 | 2 |
| **3177** | 321 | E | 6 | 50 | 19 | 1 | 2 | 2 |
| **3178** | 35 | D | 2 | 10 | 8 | 1 | 4 | 17 |
| **3179** | 294 | H | 35 | 12 | 5 | 1 | 17 | 8 |
| **3181** | 262 | E | 6 | 9 | 2 | 7 | 2 | 2 |
| **3182** | 266 | D | 2 | 10 | 4 | 1 | 4 | 5 |
| **3183** | 86 | E | 6 | 1 | 2 | 6 | 14 | 2 |
| **3184** | 305 | E | 6 | 25 | 2 | 6 | 2 | 2 |
| **3185** | 12b | D | 2 | 5 | 19 | 7 | 1 | 4 |
| **3186** | 300 | F | 24 | 14 | 4 | 5 | 1 | 4 |
| **3189** | 90 | F | 24 | 5 | 19 | 1 | 1 | 4 |
| **3191** | 56b | E | 58 | 1 | 19 | 7 | 2 | 1 |
| **3192** | 157 | E | 6 | 5 | 19 | 7 | 1 | 1 |
| **3194** | 295 | J | 20 | 12 | 5 | 2 | 2 | 18 |
| **3196** | 171 | E | 6 | 49 | 19 | 7 | 2 | 1 |
| **3197** | 255 | E | 6 | 7 | 52 | 7 | 2 | 1 |
| **3198** | 340 | K | 12 | 69 | 53 | 22 | 3 | 8 |
| **3199** | 35 | D | 2 | 10 | 8 | 1 | 4 | 17 |
| **3200** | 56a | E | 6 | 1 | 19 | 7 | 2 | 1 |
| **3202** | 101 | I | 36 | 38 | 5 | 12 | 7 | 18 |
| **3203** | 256 | J | 53 | 8 | 4 | 1 | 1 | 6 |
| **3204** | 32 | K | 12 | 10 | 7 | 1 | 4 | 8 |
| **3205** | 175a | K | 12 | 10 | 6 | 22 | 3 | 8 |
| **3207** | 252 | E | 6 | 7 | 19 | 7 | 1 | 1 |
| **3208** | 289 | D | 1 | 10 | 8 | 34 | 1 | 4 |
| **3215** | 135b | I | 36 | 10 | 5 | 12 | 4 | 18 |
| **3216** | 12d | F | 24 | 5 | 19 | 7 | 1 | 4 |
| **3217** | 101 | I | 36 | 38 | 5 | 12 | 7 | 18 |
| **3218** | 240 | F | 24 | 5 | 19 | 12 | 1 | 4 |
| **3219** | 172 | E | 6 | 1 | 2 | 7 | 2 | 2 |
| **3220** | 62b | F | 24 | 5 | 19 | 7 | 3 | 4 |
| **3221** | 225 | E | 6 | 1 | 19 | 6 | 2 | 1 |
| **3222** | 12d | F | 24 | 5 | 19 | 7 | 1 | 4 |
| **3223** | 271 | I | 36 | 10 | 5 | 12 | 4 | 34 |
| **3224** | 295 | J | 20 | 12 | 5 | 2 | 2 | 18 |
| **3225** | 94 | G | 8 | 10 | 5 | 12 | 3 | 5 |
| **3227** | 130b | G | 8 | 10 | 4 | 1 | 3 | 5 |
| **3228** | 100b | I | 36 | 10 | 5 | 12 | 7 | 18 |
| **3229** | 59 | E | 6 | 7 | 19 | 7 | 2 | 1 |
| **3230** | 56a | E | 6 | 1 | 19 | 7 | 2 | 1 |
| **3232** | 236 | E | 6 | 5 | 19 | 7 | 2 | 1 |
| **3233** | 276 | I | 36 | 10 | 5 | 12 | 27 | 18 |
| **3234** | 12d | F | 24 | 5 | 19 | 7 | 1 | 4 |
| **3236** | 135b | I | 36 | 10 | 5 | 12 | 4 | 18 |
| **3237** | 327 | E | 6 | 57 | 19 | 7 | 2 | 1 |
| **3238** | 247 | D | 1 | 5 | 20 | 36 | 2 | 34 |
| **3239** | 333 | F | 24 | 63 | 19 | 5 | 2 | 4 |
| **3241** | 12d | F | 24 | 5 | 19 | 7 | 1 | 4 |
| **3242** | 100b | I | 36 | 10 | 5 | 12 | 7 | 18 |
| **3244** | 27 | G | 9 | 10 | 6 | 10 | 1 | 6 |
| **3245** | 257 | K | 12 | 8 | 4 | 1 | 7 | 8 |
| **3246** | 56a | E | 6 | 1 | 19 | 7 | 2 | 1 |
| **3247** | 174 | E | 6 | 5 | 19 | 7 | 1 | 2 |
| **3248** | 174 | E | 6 | 5 | 19 | 7 | 1 | 2 |
| **3251** | 226 | E | 6 | 1 | 19 | 7 | 2 | 61 |
| **3252** | 286 | D | 62 | 10 | 8 | 22 | 3 | 17 |
| **3253** | 101 | I | 36 | 38 | 5 | 12 | 7 | 18 |
| **3254** | 270 | G | 8 | 10 | 5 | 12 | 3 | 8 |
| **3256** | 242 | D | 1 | 5 | 20 | 5 | 2 | 2 |
| **3257** | 12d | F | 24 | 5 | 19 | 7 | 1 | 4 |
| **3259** | 12a | D | 1 | 5 | 19 | 7 | 1 | 4 |
| **3260** | 3 | E | 6 | 1 | 2 | 6 | 2 | 2 |
| **3261** | 56a | E | 6 | 1 | 19 | 7 | 2 | 1 |
| **3262** | 64a | E | 6 | 4 | 19 | 7 | 2 | 1 |
| **3263** | 12d | F | 24 | 5 | 19 | 7 | 1 | 4 |
| **3264** | 296 | G | 8 | 12 | 5 | 11 | 9 | 5 |
| **3266** | 286 | D | 62 | 10 | 8 | 22 | 3 | 17 |
| **3268** | 12a | D | 1 | 5 | 19 | 7 | 1 | 4 |
| **3269** | 56a | E | 6 | 1 | 19 | 7 | 2 | 1 |
| **3270** | 231 | F | 24 | 5 | 19 | 1 | 2 | 4 |
| **3271** | 296 | G | 8 | 12 | 5 | 11 | 9 | 5 |
| **3272** | 197b | F | 24 | 1 | 2 | 7 | 2 | 6 |
| **3273** | 3 | E | 6 | 1 | 2 | 6 | 2 | 2 |
| **3274** | 109 | D | 1 | 5 | 20 | 5 | 2 | 34 |
| **3276** | 174 | E | 6 | 5 | 19 | 7 | 1 | 2 |
| **3277** | 338 | E | 6 | 67 | 2 | 7 | 2 | 60 |
| **3278** | 312 | D | 1 | 35 | 19 | 7 | 2 | 10 |
| **3279** | 238b | F | 24 | 5 | 19 | 7 | 15 | 4 |
| **3281** | 338 | E | 6 | 67 | 2 | 7 | 2 | 60 |
| **3283** | 238a | D | 1 | 5 | 19 | 7 | 15 | 4 |
| **3284** | 12e | J | 38 | 5 | 19 | 7 | 1 | 4 |
| **3285** | 90 | F | 24 | 5 | 19 | 1 | 1 | 4 |
| **3286** | 16 | E | 6 | 7 | 19 | 14 | 2 | 1 |
| **3288** | 327 | E | 6 | 57 | 19 | 7 | 2 | 1 |
| **3289** | 283 | D | 2 | 10 | 8 | 10 | 1 | 17 |
| **3292** | 12d | F | 24 | 5 | 19 | 7 | 1 | 4 |
| **3293** | 77b | D | 31 | 5 | 19 | 7 | 2 | 37 |
| **3295** | 12d | F | 24 | 5 | 19 | 7 | 1 | 4 |
| **3297** | 348 | J | 20 | 77 | 5 | 12 | 4 | 18 |
| **3299** | 99 | J | 38 | 36 | 15 | 7 | 1 | 4 |
| **3300** | 32 | K | 12 | 10 | 7 | 1 | 4 | 8 |
| **3301** | 3 | E | 6 | 1 | 2 | 6 | 2 | 2 |
| **3302** | 101 | I | 36 | 38 | 5 | 12 | 7 | 18 |
| **3303** | 69 | E | 6 | 5 | 19 | 6 | 2 | 2 |
| **3304** | 299 | F | 24 | 13 | 19 | 12 | 2 | 4 |
| **3306** | 101 | I | 36 | 38 | 5 | 12 | 7 | 18 |
| **3307** | 279 | I | 37 | 10 | 7 | 1 | 4 | 5 |
| **3308** | 315 | I | 36 | 38 | 5 | 12 | 27 | 18 |
| **3309** | 135b | I | 36 | 10 | 5 | 12 | 4 | 18 |
| **3310** | 342 | E | 6 | 71 | 2 | 7 | 2 | 2 |
| **3311** | 264 | J | 53 | 10 | 4 | 1 | 1 | 6 |
| **3312** | 326 | E | 60 | 57 | 2 | 6 | 2 | 2 |
| **3313** | 62a | D | 1 | 5 | 19 | 7 | 3 | 4 |
| **3314** | 62a | D | 1 | 5 | 19 | 7 | 3 | 4 |
| **3319** | 30 | K | 12 | 10 | 7 | 1 | 3 | 8 |
| **3320** | 265 | G | 10 | 10 | 4 | 1 | 3 | 7 |
| **3321** | 56a | E | 6 | 1 | 19 | 7 | 2 | 1 |
| **3324** | 281a | J | 20 | 10 | 8 | 1 | 4 | 18 |
| **3325** | 12d | F | 24 | 5 | 19 | 7 | 1 | 4 |
| **3327** | 12d | F | 24 | 5 | 19 | 7 | 1 | 4 |
| **3328** | 3 | E | 6 | 1 | 2 | 6 | 2 | 2 |
| **3329** | 100b | I | 36 | 10 | 5 | 12 | 7 | 18 |
