# Supplementary material for: The Role of Surinamese Migrants in the Transmission of Chlamydia trachomatis between Paramaribo, Suriname and Amsterdam, The Netherlands
Source: PLoS One. 2013 Nov 13;8(11):e77977. doi: 10.1371/journal.pone.0077977 (PMC3827209; doi:10.1371/journal.pone.0077977)
Supplement: Table S2 — Characteristics of Chlamydia trachomatis -positive participants, by C. trachomatis cluster, from Paramaribo, Suriname and Amsterdam, the Netherlands, 2008–10. (DOC) [file pone.0077977.s003.doc]

*Table S2. Characteristics of Chlamydia trachomatis-positive participants, by C. trachomatis cluster, from Paramaribo, Suriname and Amsterdam, the Netherlands, 2008-10.*

|  |  | **Cluster 1**  **(n=105)** | **Cluster 2**  **(n=76)** | **Cluster 3**  **(n=62)** | **Cluster 4**  **(n=53)** | **Residual group**  **(n=130)** | ***p*** |
| --- | --- | --- | --- | --- | --- | --- | --- |
|  |  | **n (%)** | **n (%)** | **n (%)** | **n (%)** | **n (%)** |  |
| **Gender** | Male | 31 (30) | 31 (41) | 17 (27) | 26 (49) | 46 (35) | **0.07** |
|  | Female | 74 (70) | 45 (59) | 45 (73) | 27 (51) | 84 (65) |  |
| **Age in years** | Median (mean; IQR) | 24 (24.6; 21-26) | 24 (24.4; 21-26) | 24 (26.8; 22-30) | 24 (26.2; 21-29) | 24 (26.8; 21-29) | **0.27** |
| **Education^a^** | Low | 14 (14) | 3 (4) | 17 (28) | 12 (23) | 24 (19) | **0.003** |
|  | Medium | 53 (51) | 34 (45) | 25 (41) | 28 (54) | 56 (45) |  |
|  | High | 36 (35) | 39 (51) | 19 (31) | 12 (23) | 45 (36) |  |
| **Ethnic group^b^** | Native Surinamese | 38 (37) | 9 (12) | 36 (59) | 25 (47) | 47 (37) | **<0.001** |
|  | Native Dutch | 40 (39) | 48 (64) | 16 (26) | 6 (11) | 56 (44) |  |
|  | Dutch Migrant | 1 (1) | 0 (0) | 0 (0) | 0 (0) | 1 (1) |  |
|  | Surinamese Migrant | 15 (15) | 11 (15) | 5 (8) | 20 (38) | 13 (10) |  |
|  | Other | 9 (9) | 7 (9) | 4 (7) | 2 (4) | 11 (9) |  |
| **Number of sexual partners in the past 12 months^c^** | Median (mean; IQR) | 1 (1.5; 1-2) | 1 (2.5; 1-2) | 1 (1.3; 1-1) | 1 (1.7; 1-2) | 1 (1.8; 1-2) | **0.13** |

*^a^ Data were missing for 2 participants in Cluster 1, 1 participant in Cluster 3, 1 participant in Cluster 4 and 5 participants in Residual group.*

*^b^ Data were missing for 2 participants in Cluster 1, 1 participant in Cluster 2, 1 participant in Cluster 3 and 2 participants in Residual group.*

*^c^ Data were missing for 1 participant in Cluster 2, 2 participants in Cluster 3 and 3 participants in Residual group.*

IQR: interquartile range
