# Supplementary material for: The Role of Surinamese Migrants in the Transmission of Chlamydia trachomatis between Paramaribo, Suriname and Amsterdam, The Netherlands
Source: PLoS One. 2013 Nov 13;8(11):e77977. doi: 10.1371/journal.pone.0077977 (PMC3827209; doi:10.1371/journal.pone.0077977)
Supplement: Table S3 — Characteristics of Chlamydia trachomatis -positive participants, by C. trachomatis cluster. From Paramaribo, Suriname, 2008–10 (A), and from Amsterdam, the Netherlands, 2009–10 (B). (DOCX) [file pone.0077977.s004.docx]

*Table S3A. Characteristics of Chlamydia trachomatis-positive participants, by C. trachomatis cluster, from Paramaribo, Suriname, 2008-10.*

|  |  | **Cluster 1**  **(n=43)** | **Cluster 2**  **(n=9)** | **Cluster 3**  **(n=37)** | **Cluster 4**  **(n=27)** | **Residual group**  **(n=54)** | ***p*** |
| --- | --- | --- | --- | --- | --- | --- | --- |
|  |  | **n (%)** | **n (%)** | **n (%)** | **n (%)** | **n (%)** |  |
| **Gender** | Male | 15 (35) | 2 (22) | 10 (27) | 16 (59) | 22 (41) | **0.08** |
|  | Female | 28 (65) | 7 (78) | 27 (73) | 11 (41) | 32 (59) |  |
| **Age in years** | Median (mean; IQR) | 25 (26.7; 22-31) | 26 (30.1; 25-30) | 25 (27.5; 23-31) | 26 (27.5; 21-33) | 24 (26.2; 21-28) | **0.54** |
| **Education^a^** | Low | 14 (33) | 2 (22) | 17 (46) | 12 (46) | 23 (45) | **0.19** |
|  | Medium | 19 (45) | 5 (56) | 15 (41) | 10 (38) | 27 (53) |  |
|  | High | 9 (21) | 2 (22) | 5 (14) | 4 (15) | 1 (2) |  |
| **Ethnic group^b^** | Native Surinamese | 38 (90) | 9 (100) | 36 (97) | 25 (93) | 47 (89) | **0.99** |
|  | Native Dutch | - | - | - | - | - |  |
|  | Dutch Migrant | 1 (2) | 0 (0) | 0 (0) | 0 (0) | 1 (2) |  |
|  | Surinamese Migrant | 1 (2) | 0 (0) | 0 (0) | 1 (4) | 2 (4) |  |
|  | Other | 2 (5) | 0 (0) | 1 (3) | 1 (4) | 3 (6) |  |
| **Number of sexual partners in the past 12 months^c^** | Median (mean; IQR) | 1 (1.4; 1-1) | 1 (1.4; 1-2) | 1 (1.2; 1-1) | 1 (1.3; 1-2) | 1 (1.6; 1-2) | **0.34** |

*^a^ Data were missing for 1 participant in Cluster 1, 1 participant in Cluster 4 and 3 participants in Residual group.*

*^b^ Data were missing for 1 participant in Cluster 1 and 1 participant in Residual group.*

*^c^ Data were missing for 2 participants in Cluster 3 and 3 participants in Residual group.*

IQR: interquartile range

*Table S3B. Characteristics of Chlamydia trachomatis-positive participants, by C. trachomatis cluster, from Amsterdam, the Netherlands, 2009-10.*

|  |  | **Cluster 1**  **(n=62)** | **Cluster 2**  **(n=67)** | **Cluster 3**  **(n=25)** | **Cluster 4**  **(n=26)** | **Residual group**  **(n=76)** | ***p*** |
| --- | --- | --- | --- | --- | --- | --- | --- |
|  |  | **n (%)** | **n (%)** | **n (%)** | **n (%)** | **n (%)** |  |
| **Gender** | Male | 16 (26) | 29 (43) | 7 (28) | 10 (38) | 24 (32) | **0.26** |
|  | Female | 46 (74) | 38 (57) | 18 (72) | 16 (62) | 52 (68) |  |
| **Age in years** | Median (mean; IQR) | 22 (23.2; 20-25) | 23 (23.6; 21-25) | 24 (25.8; 21-28) | 24 (24.7; 21-26) | 23 (27.2; 20-31) | **0.34** |
| **Education^a^** | Low | 0 (0) | 1 (1) | 0 (0) | 0 (0) | 1 (1) | **0.12** |
|  | Medium | 34 (56) | 29 (43) | 10 (42) | 18 (69) | 29 (39) |  |
|  | High | 27 (44) | 37 (55) | 14 (58) | 8 (31) | 44 (59) |  |
| **Ethnic group^b^** | Native Surinamese | - | - | - | - | - | **<0.001** |
|  | Native Dutch | 40 (66) | 48 (73) | 16 (67) | 6 (23) | 56 (75) |  |
|  | Dutch Migrant | - | - | - | - | - |  |
|  | Surinamese Migrant | 14 (23) | 11 (17) | 5 (21) | 19 (73) | 11 (15) |  |
|  | Other | 7 (11) | 7 (11) | 3 (13) | 1 (4) | 8 (11) |  |
| **Number of sexual partners in the past 12 months^c^** | Median (mean; IQR) | 1 (1.5; 1-2) | 1 (2.7; 1-2) | 1 (1.4; 1-2) | 1 (2.2; 1-2) | 1 (2.0; 1-2) | **0.75** |

*^a^ Data were missing for 1 participant in Cluster 1, 1 participant in Cluster 3 and 2 participants in Residual group.*

*^b^ Data were missing for 1 participant in Cluster 1, 1 participant in Cluster 2, 1 participant in Cluster 3 and 1 participant in Residual group.*

*^c^ Data were missing for 1 participant in Cluster 2.*

IQR: interquartile range
