# Supplementary material for: The Role of Surinamese Migrants in the Transmission of Chlamydia trachomatis between Paramaribo, Suriname and Amsterdam, The Netherlands
Source: PLoS One. 2013 Nov 13;8(11):e77977. doi: 10.1371/journal.pone.0077977 (PMC3827209; doi:10.1371/journal.pone.0077977)
Supplement: Table S4 — Characteristics of Chlamydia trachomatis-positive native Surinamese participants, by C. trachomatis cluster, 2008–10 (A). Characteristics of Chlamydia trachomatis-positive native Dutch participants, by C. trachomatis cluster, 2009–10 (B). Characteristics of Chlamydia trachomatis-positive Surinamese migrant participants, by C. trachomatis cluster, 2009–10 (C). (DOCX) [file pone.0077977.s005.docx]

*Table S4A. Characteristics of trachomatis-positive native Surinamese participants, by C. trachomatis cluster, 2008-10.*

|  |  | **Cluster 1**  **(n=38)** | **Cluster 2**  **(n=9)** | **Cluster 3**  **(n=36)** | **Cluster 4**  **(n=25)** | **Residual group**  **(n=47)** | ***p*** |
| --- | --- | --- | --- | --- | --- | --- | --- |
|  |  | **n (%)** | **n (%)** | **n (%)** | **n (%)** | **n (%)** |  |
| **Gender** | Male | 13 (34) | 2 (22) | 10 (28) | 15 (60) | 19 (40) | **0.09** |
|  | Female | 25 (66) | 7 (78) | 26 (72) | 10 (40) | 28 (60) |  |
| **Age in years** | Median (mean; IQR) | 26 (27.5; 24-31) | 26 (30.1; 25-30) | 26 (27.7; 23-32) | 26 (27.4; 21-33) | 24 (25.9; 21-28) | **0.36** |
| **Education^a^** | Low | 12 (32) | 2 (22) | 16 (44) | 12 (50) | 21 (47) | **0.31** |
|  | Medium | 19 (50) | 5 (56) | 15 (42) | 9 (38) | 23 (51) |  |
|  | High | 7 (18) | 2 (22) | 5 (14) | 3 (13) | 1 (2) |  |
| **Number of sexual partners in the past 12 months^b^** | Median (mean; IQR) | 1 (1.4; 1-1) | 1 (1.4; 1-2) | 1 (1.2; 1-1) | 1 (1.3; 1-2) | 1 (1.6; 1-2) | **0.50** |

*^a^ Data were missing for 1 participant in Cluster 4 and 2 participants in Residual group.*

*^b^ Data were missing for 1 participant in Cluster 3.*

IQR: interquartile range

*Table S4B. Characteristics of trachomatis-positive native Dutch participants, by C. trachomatis cluster, 2009-10.*

|  |  | **Cluster 1**  **(n=40)** | **Cluster 2**  **(n=48)** | **Cluster 3**  **(n=16)** | **Cluster 4**  **(n=6)** | **Residual group**  **(n=56)** | ***p*** |
| --- | --- | --- | --- | --- | --- | --- | --- |
|  |  | **n (%)** | **n (%)** | **n (%)** | **n (%)** | **n (%)** |  |
| **Gender** | Male | 9 (23) | 18 (38) | 4 (25) | 3 (50) | 18 (32) | **0.47** |
|  | Female | 31 (78) | 30 (63) | 12 (75) | 3 (50) | 38 (68) |  |
| **Age in years** | Median (mean; IQR) | 22 (23.1; 20-25) | 22 (23.6; 20-24) | 24 (23.6; 20-26) | 20 (21.0; 19-24) | 24 (28.3; 21-31) | **0.07** |
| **Education** | Low | - | - | - | - | - | **0.12** |
|  | Medium | 17 (43) | 18 (38) | 5 (31) | 5 (83) | 17 (30) |  |
|  | High | 23 (58) | 30 (63) | 11 (69) | 1 (17) | 39 (70) |  |
| **Number of sexual partners in the past 12 months** | Median (mean; IQR) | 1 (1.5; 1-2) | 1 (2.0; 1-2) | 1 (1.3; 1-2) | 1 (1.8; 1-3) | 1 (2.1; 1-2) | **0.47** |

IQR: interquartile range

*Table S4C. Characteristics of trachomatis-positive Surinamese migrant participants, by C. trachomatis cluster, 2009-10.*

|  |  | **Cluster 1**  **(n=15)** | **Cluster 2**  **(n=11)** | **Cluster 3**  **(n=5)** | **Cluster 4**  **(n=20)** | **Residual group**  **(n=13)** | ***p*** |
| --- | --- | --- | --- | --- | --- | --- | --- |
|  |  | **n (%)** | **n (%)** | **n (%)** | **n (%)** | **n (%)** |  |
| **Gender** | Male | 6 (40) | 7 (64) | 2 (40) | 8 (40) | 7 (54) | **0.69** |
|  | Female | 9 (60) | 4 (36) | 3 (60) | 12 (60) | 6 (46) |  |
| **Age in years** | Median (mean; IQR) | 21 (20.7; 19-25) | 23 (22.4; 20-24) | 25 (28.4; 21-38) | 25 (25.9; 22-29) | 23 (25.5; 19-29) | **0.11** |
| **Education^a^** | Low | 0 (0) | 0 (0) | 0 (0) | 0 (0) | 2 (17) | **0.38** |
|  | Medium | 12 (86) | 7 (64) | 4 (80) | 13 (65) | 8 (67) |  |
|  | High | 2 (14) | 4 (36) | 1 (20) | 7 (35) | 2 (17) |  |
| **Number of sexual partners in the past 12 months^b^** | Median (mean; IQR) | 1 (1.8; 1-2) | 2 (1.7; 1-2) | 1 (1.4; 1-2) | 1 (2.3; 1-3) | 1 (1.7; 1-2) | **0.99** |

*^a^ Data were missing for 1 participant in Cluster 1 and 1 participant in Residual group.*

*^b^ Data were missing for 1 participant in Cluster 2.*

IQR: interquartile range
